# Uplift modeling to identify patients who require extensive catheter ablation procedures among patients with persistent atrial fibrillation

**DOI:** 10.1038/s41598-024-52976-7

**Published:** 2024-02-01

**Authors:** Taiki Sato, Yohei Sotomi, Shungo Hikoso, Tetsuhisa Kitamura, Daisaku Nakatani, Katsuki Okada, Tomoharu Dohi, Akihiro Sunaga, Hirota Kida, Yuki Matsuoka, Nobuaki Tanaka, Tetsuya Watanabe, Nobuhiko Makino, Yasuyuki Egami, Takafumi Oka, Hitoshi Minamiguchi, Miwa Miyoshi, Masato Okada, Takashi Kanda, Yasuhiro Matsuda, Masato Kawasaki, Masaharu Masuda, Koichi Inoue, Yasushi Sakata, the OCVC-Arrhythmia Investigators, Toshiaki Mano, Masatake Fukunami, Takahisa Yamada, Yoshio Furukawa, Shinji Hasegawa, Yoshiharu Higuchi, Akio Hirata, Jun Tanouchi, Masami Nishino, Yasuharu Matsunaga, Yasushi Matsumura, Hiroya Mizuno, Toshihiro Takeda, Tomoaki Nakano, Kentaro Ozu, Shinichiro Suna, Bolrathanak Oeun, Koji Tanaka, Tomoko Minamisaka, Shiro Hoshida

**Affiliations:** 1 Department of Cardiovascular Medicine, Osaka University Graduate School of Medicine, 2-2, Yamadaoka, Suita, Osaka 565-0871 Japan; 2 Department of Social and Environmental Medicine, Osaka University Graduate School of Medicine, 2-2, Yamadaoka, Suita, Osaka 565-0871 Japan; 3 Department of Transformative System for Medical Information, Osaka University Graduate School of Medicine, 2-2, Yamadaoka, Suita, Osaka 565-0871 Japan; 4 Cardiovascular Center, Sakurabashi Watanabe Hospital, Osaka, Japan; 5 Division of Cardiology, Osaka General Medical Center, Osaka, Japan; 6 Department of Cardiovascular Medicine, Yao Municipal Hospital, Yao, Japan; 7 Cardiovascular Division, Osaka Police Hospital, Osaka, Japan; 8 Division of Cardiology, Osaka Rosai Hospital, Sakai, Japan; 9 Department of Cardiology, Osaka Hospital, Japan Community Healthcare Organization, Osaka, Japan; 10 Cardiovascular Center, Kansai Rosai Hospital, Amagasaki, Japan; 11 Cardiovascular Division, National Hospital Organization Osaka National Hospital, Osaka, Japan; 12 Yao Municipal Hospital, Yao, Japan

**Keywords:** Interventional cardiology, Atrial fibrillation

## Abstract

Identifying patients who would benefit from extensive catheter ablation along with pulmonary vein isolation (PVI) among those with persistent atrial fibrillation (AF) has been a subject of controversy. The objective of this study was to apply uplift modeling, a machine learning method for analyzing individual causal effect, to identify such patients in the EARNEST-PVI trial, a randomized trial in patients with persistent AF. We developed 16 uplift models using different machine learning algorithms, and determined that the best performing model was adaptive boosting using Qini coefficients. The optimal uplift score threshold was 0.0124. Among patients with an uplift score ≥ 0.0124, those who underwent extensive catheter ablation (PVI-plus) showed a significantly lower recurrence rate of AF compared to those who received only PVI (PVI-alone) (HR 0.40; 95% CI 0.19–0.84; *P*-value = 0.015). In contrast, among patients with an uplift score < 0.0124, recurrence of AF did not significantly differ between PVI-plus and PVI-alone (HR 1.17; 95% CI 0.57–2.39; *P*-value = 0.661). By employing uplift modeling, we could effectively identify a subset of patients with persistent AF who would benefit from PVI-plus. This model could be valuable in stratifying patients with persistent AF who need extensive catheter ablation before the procedure.

## Introduction

Utilizing machine learning presents a promising approach to enhance the diagnosis and treatment of various ailments, such as cardiovascular diseases. Specifically, appropriate stratification and patient selection are crucial steps towards administering effective treatment. Uplift modeling^1^, a common machine learning methodology used in commercial industries to discern individuals with a greater or lesser propensity to purchase a product in response to an intervention, can also facilitate the identification of patients who would benefit most from treatment. This technique is unlike traditional statistical analysis, which usually aims to determine whether a treatment is effective overall or whether the effectiveness of the treatment differs among a small number of pre-defined subgroups. Uplift models are frequently trained using outcome data in the form of a customer’s response to an intervention. Randomized clinical trial data can also be utilized to train uplift models to identify patients who would benefit from a particular treatment^2^.

Catheter ablation presents itself as a secure and efficacious treatment for atrial fibrillation (AF). While pulmonary vein isolation (PVI) is typically performed on AF patients^3,4^ it exhibits inadequate efficacy in maintaining sinus rhythm among individuals with persistent AF as opposed to those with paroxysmal AF. Despite the integration of extensive catheter ablation, such as linear ablation and/or complex fractional atrial electrogram (CFAE) ablation, with PVI for individuals with persistent AF^5^, randomized clinical trials evaluating the efficacy of this combination treatment have failed to produce definitive results, and the efficacy of extensive ablation remains a topic of controversy^6,7^. This may be attributed to the heterogeneity of patients with persistent AF, thus highlighting the importance of suitable stratification and identification of individuals who do or do not require extensive substrate ablation in conjunction with PVI. Though numerous previous studies have delved into this issue, including our own^8–11^, the optimal method for stratification has yet to be elucidated. In this regard, uplift modeling would be an appropriate approach.

Here, we report the usefulness of uplift modeling in identifying patients who necessitate extensive catheter ablation along with PVI among those afflicted with persistent AF. Moreover, we detail the features of patients identified by uplift modeling who stand to benefit from extensive ablation.

## Methods

### Study design

This study was a post-hoc sub-analysis of the EARNEST-PVI trial, registered at ClinicalTrials.gov (https://clinicaltrials.gov/ct2/show/NCT03514693, ClinicalTrials.gov Identifier: NCT03514693)^7,9,10,12,13^, which focused on stratification using uplift modeling. The EARNEST-PVI trial is a prospective, multicenter, randomized, and open-label non-inferiority trial of patients with persistent AF undergoing an initial catheter ablation procedure conducted by the Osaka Cardiovascular Conference Arrhythmia Investigators. Details of the EARNEST-PVI trial are described elsewhere^7,12^. Briefly, patients with persistent AF undergoing a first-time ablation procedure were enrolled in eight medical centers with extensive experience with catheter ablation for AF. Patients were randomized to receive either PVI only (PVI-alone) or extensive ablation comprising linear and/or CFAE ablation in addition to PVI (PVI-plus). Before catheter ablation, we collected clinical data, including patient history, laboratory data, and transthoracic echocardiography results. Details of the ablation procedures are also described elsewhere^7,12^. Patients were followed up for 12 months after the ablation procedure. A 12-lead electrocardiogram (ECG) was performed before catheter ablation, at discharge, and at 1, 3, 6, 9, and 12 months, and a 24-h Holter ECG was conducted at 6 and 12 months to detect recurrence of AF. The primary endpoint of the study was the recurrence of AF documented by scheduled or symptom-driven ECG tests during the 12-month follow-up period. All patients provided written informed consent to participate and the study was approved by the ethics committee of each hospital. This study conformed to the ethical guidelines outlined in the Declaration of Helsinki, and was approved by the Institutional Review Boards of all hospitals. The following institutes approved this study: Cardiovascular Center, Sakurabashi-Watanabe Hospital (study number: 17-6); Osaka University Graduate School of Medicine (14377); Kansai Rosai Hospital (15D059g); Osaka General Medical Center (27-2035); Osaka Police Hospital (548); Osaka Rosai Hospital (28-78); Yao Municipal Hospital: (八病H29-5); and Osaka Hospital, Japan Community Healthcare Organization (2016-25).

### Uplift modeling

Uplift modeling has been used to predict the difference between class probabilities in a treatment and a control group. This approach enables the discovery of a group of patients for which a treatment is more beneficial^2^.

A total of 53 variables before catheter ablation (Supplementary information 2, Supplementary Table S1) were considered as primary candidates for uplift modeling after excluding factors with missing data of more than 15%. Missing values in categorical features were imputed with a constant ‘not available’ value and those in continuous features were imputed with the mean value of the feature. This imputation method is the default preprocessing method in ‘PyCaret’ 2.3.10, an open-source, low-code machine learning library in Python (https://pycaret.readthedocs.io/en/stable/index.html). Variables with a correlation coefficient greater than 0.7 or considered clinically highly relevant to each other were removed and replaced with the variable that was considered the most informative. Continuous variables were scaled and translated according to the interquartile range in a normal distribution. Finally, a total of 26 variables were included in the present analysis (Supplementary information 2, Supplementary Table S2).

Uplift modeling is commonly conducted using a two-model approach or a one-model approach^2^. Here, we used the one-model approach. The advantage of the one-model approach is that models are easier to interpret. The predictive power of the influence of each variable on the uplift model can easily be evaluated. *X*_*i*_ is defined as a predictor variable and *Y* $\in \left\{0, 1\right\}$ as a class variable whose behavior is to be modeled. The uplift score is calculated by subtracting the probability of being assigned to the control group (${P}_{C}$) from the probability of being assigned to the treatment group (${P}_{T}$). For the class variable, a 1 value indicates a positive outcome (success) while a 0 value indicates a negative outcome (failure). In the present study, success was defined as no recurrence of AF during the 1-year follow-up period, and failure was defined as recurrence of AF during the 1-year follow-up period.

$$Uplift\; score = P_{T} (Y = 1|X_{i} ) - P_{C} (Y = 1|X_{i} )$$

The one-model approach uses class variable transformation. The model defines a target variable *Z* as follows:

$$Z = \left\{ {\begin{array}{*{20}l} {1,} \hfill & {if\;treatment \;group \;and \;success\; in\; procedure} \hfill \\ {1,} \hfill & {if\; control \;group \;and\;failure \;in\; procedure} \hfill \\ {0,} \hfill & {otherwise} \hfill \\ \end{array} } \right.$$

Using a probability of event *Z* = 1 ($P\left(Z=1|{X}_{i}\right)$), the uplift score can be calculated as follows^2^:

$$P_{T} \left( {Y = 1{|}X_{i} } \right) - P_{C} \left( {Y = 1{|}X_{i} } \right) = 2P\left( {Z = 1{|}X_{i} } \right) - 1$$

The transformation method above makes two assumptions. First, procedure allocation is independent of *X*_*i*_. Second, the probability of being assigned to the treatment group is equal to the probability of being assigned to the control group. These assumptions hold in the present study because patients in the EARNEST-PVI trial were randomly allocated to the treatment group (PVI-plus) or control group (PVI-alone) in a 1:1 ratio.

We tested the following probabilistic classification models for uplift modeling: logistic regression, random forest, k-nearest neighbor algorithm, quadratic discriminant analysis, naive Bayes, adaptive boosting, decision tree, gradient boosting, linear discriminant analysis, radial basis kernel function of support vector machine, extra trees classifier, extreme gradient boosting, Gaussian process, light gradient boosting, multi-level perceptron, and category boosting.

To evaluate each model’s diagnostic performance, we used a Qini curve because we focused on the efficacy of treatment (PVI-plus). The model returns an uplift score for each individual, and the data are sorted in descending order. The Qini curve plots the incremental success, which is calculated by

$$Qini\;curve\left( \varphi \right) = S_{T} \left( \varphi \right) - \frac{{S_{C} \left( \varphi \right)N_{T} \left( \varphi \right)}}{{N_{C} \left( \varphi \right)}}$$

where ${S}_{T}(\varphi )$ and ${S}_{C}(\varphi )$ are the number of cumulative successes in individuals with an uplift score ≥ $\varphi$ in the treatment group and control group, respectively. ${N}_{T}(\varphi )$ and ${N}_{C}(\varphi )$ are the number of individuals with an uplift score ≥ $\varphi$ in the treatment group and control group, respectively. In a graph on Qini curve, the horizontal axis shows ranking of uplift score sorted in descending order, not uplift score in itself, and the vertical axis shows cumulative uplift. Finally, the Qini coefficient is calculated by measuring the area between the Qini curve and the diagonal line. The diagonal line represents incremental success if the treatment is randomly allocated. A model with a higher Qini coefficient has higher diagnostic performance. The optimal cut-off value is the uplift score of an individual with a maximum score of $\varphi$, calculated by subtracting incremental success on $Qini curve\left(\varphi \right)$ from that on the diagonal line. After applying each model to the validation cohort of training dataset, we selected the one with the highest Qini coefficient as the best performing model. Gini importance was used to rank the importance of the features in the selected model where possible in the cohort of the training dataset for model selection^14^, which is the default setting in the ‘PyCaret’ package. In addition, we used SHapley Additive exPlanations (SHAP) methodology to speculate impact on model output according to each of the variables in training, model selection, and test^15^. The SHAP approach enables the identification and prioritization of features that determine compound classification and activity prediction using any machine learning model^15^.

We have included files to perform uplift score calculations in Supplementary information 3 (Supplementary materials).

### Dataset

**Figure 1:**
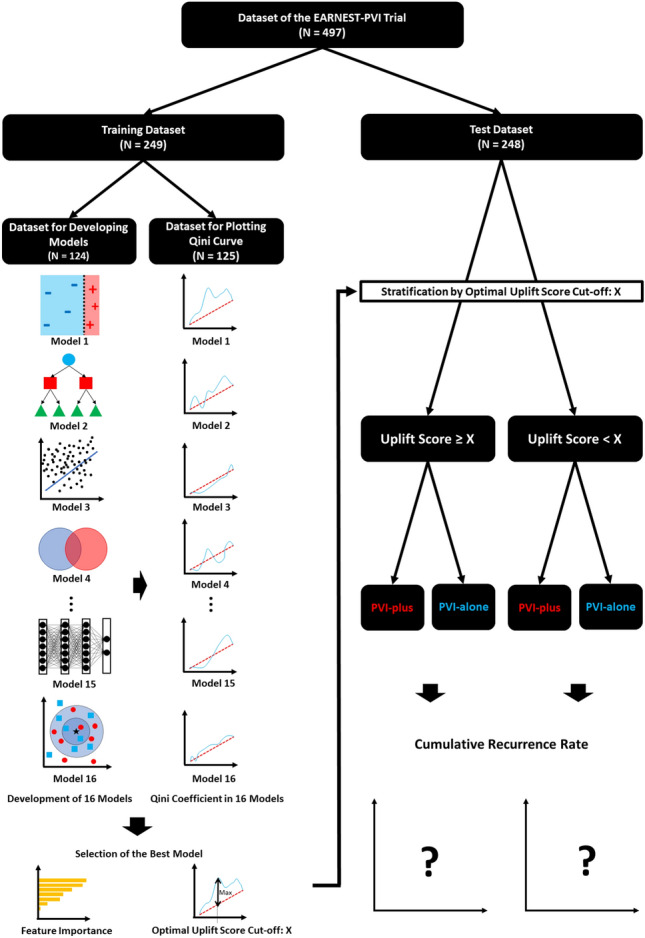
Study flow chart. PVI: pulmonary vein isolation.

We divided the EARNEST-PVI trial (N = 497) dataset in a 1:1 ratio according to patients’ order of registration. The number of patients in the training and test datasets was 249 and 248, respectively. In addition, we further divided the training dataset in a 1:1 ratio according to patients’ order of registration: one dataset (N = 124) was used to train the models and the other (N = 125) was used to calculate Qini coefficients and determine the optimal uplift score cut-off predicted by the trained models. We subsequently selected the model with the highest Qini coefficient and used the optimal uplift score cut-off to divide the test dataset into two groups. A study flowchart is shown in Fig. 1.

### Statistical analysis

Statistical analysis was conducted using Python 3.9.12 and R 4.0.5. We performed intention-to-treat analysis in this study. Continuous variables are presented as median with interquartile range (median [25th percentile, 75th percentile]) and categorical data as counts and percentages. Demographic and procedural differences were analyzed using the Mann–Whitney *U* test for continuous variables, and Fisher’s exact test for categorical variables. In Tables 1, 2, 3 and 4, continuous values are shown as median with interquartile range (median [25th percentile, 75th percentile]), and categorical values are shown as number with percentage of positive findings per number of patients studied (N (%)). *P*-values in Tables 1, and 2 were calculated by comparison between PVI-alone and PVI-plus by uplift score ≥ 0.0124 and uplift score < 0.0124. *P*-value in Table 3 was calculated by comparison between uplift score ≥ 0.0124 and uplift score < 0.0124. *P*-value in Table 4 was calculated by comparison between PVI-alone and PVI-plus by uplift score ≥ 0.0124 and uplift score < 0.0124. The cumulative event rate was calculated using the Kaplan–Meier method. The hazard ratio (HR), 95% confidence interval (CI), *P*-value, and *P*-value for interaction between the uplift score cut-off and treatment were calculated using the Cox proportional hazards model. The Kaplan–Meier method and the Cox proportional hazards model were applied to the test dataset, which was not included in process for training and model selection. The proportional hazards assumption of the treatment strategy for the primary endpoint was confirmed using Schoenfeld residuals (*P* > 0.05). *P*-values < 0.05 were used to indicate statistical significance.

**Table 1:** Patient characteristics and outcomes in the training dataset used to train models.

	PVI-alone	PVI-plus	*P*-value
N	63	61	
Age	68.00 [60.00, 72.50]	65.00 [58.00, 72.00]	0.379
Female sex	15 (23.8)	18 (29.5)	0.544
Body mass index	24.24 [21.74, 26.87]	23.32 [21.63, 26.02]	0.284
Family history of atrial fibrillation	9 (14.3)	4 (6.6)	0.241
Long-standing persistent atrial fibrillation	13 (20.6)	15 (24.6)	0.670
Hypertension	41 (65.1)	34 (55.7)	0.359
Diabetes mellitus	7 (11.1)	13 (21.3)	0.147
Dyslipidemia	30 (47.6)	30 (49.2)	> 0.999
Smoking history	46 (73.0)	37 (60.7)	0.182
Heart failure	12 (19.0)	12 (19.7)	> 0.999
Dilated cardiomyopathy	0 (0.0)	0 (0.0)	NA
Hypertrophic cardiomyopathy	0 (0.0)	1 (1.6)	0.492
Sick sinus syndrome	1 (1.6)	1 (1.6)	> 0.999
Stroke or systemic thromboembolism	4 (6.3)	6 (9.8)	0.526
Sleep apnea syndrome	6 (9.5)	8 (13.1)	0.580
Thyroid disease	2 (3.2)	3 (4.9)	0.677
Chronic obstructive pulmonary disease	5 (7.9)	1 (1.6)	0.208
Liver disease	8 (12.7)	6 (9.8)	0.778
History of use of anti-arrhythmic drug	23 (36.5)	13 (21.3)	0.076
Hemoglobin	14.70 [13.35, 15.50]	14.20 [13.50, 14.90]	0.168
Brain type natriuretic peptide	143.65 [91.30, 200.45]	130.30 [86.47, 204.15]	0.958
Creatinine	0.88 [0.75, 0.97]	0.85 [0.77, 1.01]	0.697
C-reactive protein	0.10 [0.05, 0.13]	0.10 [0.08, 0.18]	0.243
Left ventricular ejection fraction	63.60 [59.88, 69.38]	63.37 [57.07, 66.39]	0.383
Left atrial diameter	41.00 [38.70, 44.00]	41.00 [37.10, 44.60]	0.670
Mitral regurgitation	0 (0.0)	0 (0.0)	NA
Recurrence of atrial fibrillation	18 (28.6)	11 (18.0)	0.205

**Table 2:** Patient characteristics and outcomes in the training dataset used to plot Qini curves.

	PVI-alone	PVI-plus	*P*-value
N	62	63	
Age	66.00 [59.00, 72.75]	67.00 [60.00, 73.00]	0.711
Female sex	16 (25.8)	18 (28.6)	0.841
Body mass index	24.71 [22.24, 27.03]	24.77 [21.68, 26.40]	0.853
Family history of atrial fibrillation	5 (8.1)	7 (11.1)	0.763
Long-standing persistent atrial fibrillation	11 (17.7)	17 (27.0)	0.284
Hypertension	34 (54.8)	37 (58.7)	0.720
Diabetes mellitus	11 (17.7)	10 (15.9)	0.815
Dyslipidemia	32 (51.6)	29 (46.0)	0.593
Smoking history	39 (62.9)	38 (60.3)	0.855
Heart failure	13 (21.0)	14 (22.2)	> 0.999
Dilated cardiomyopathy	2 (3.2)	0 (0.0)	0.244
Hypertrophic cardiomyopathy	0 (0.0)	1 (1.6)	> 0.999
Sick sinus syndrome	1 (1.6)	1 (1.6)	> 0.999
Stroke or systemic thromboembolism	4 (6.5)	9 (14.3)	0.241
Sleep apnea syndrome	9 (14.5)	6 (9.5)	0.423
Thyroid disease	2 (3.2)	6 (9.5)	0.273
Chronic obstructive pulmonary disease	3 (4.8)	2 (3.2)	0.680
Liver disease	2 (3.2)	3 (4.8)	> 0.999
History of use of anti-arrhythmic drug	13 (21.0)	13 (20.6)	> 0.999
Hemoglobin	14.80 [14.03, 15.60]	14.20 [13.45, 14.90]	0.003
Brain type natriuretic peptide	132.20 [91.50, 208.10]	162.30 [104.70, 239.00]	0.258
Creatinine	0.89 [0.79, 0.99]	0.88 [0.76, 0.96]	0.582
C-reactive protein	0.10 [0.07, 0.19]	0.10 [0.07, 0.19]	0.924
Left ventricular ejection fraction	62.39 [55.04, 67.48]	64.04 [56.61, 68.61]	0.498
Left atrial diameter	41.60 [40.00, 44.20]	43.20 [39.90, 47.00]	0.104
Mitral regurgitation	0 (0.0)	4 (6.3)	0.119
Recurrence of atrial fibrillation	19 (30.6)	12 (19.0)	0.151

**Table 3:** Patient characteristics in three datasets.

	Training dataset used to train models	Training dataset used to plot Qini curves	Test dataset	*P-value
N	124	125	248	
Age	66.50 [58.00, 72.00]	66.00 [59.00, 73.00]	67.00 [59.00, 72.00]	0.886
Female sex	33 (26.6)	34 (27.2)	54 (21.8)	0.399
Body mass index	23.98 [21.60, 26.82]	24.73 [22.13, 26.71]	24.34 [22.29, 26.46]	0.462
Family history of atrial fibrillation	13 (10.5)	12 (9.6)	13 (5.2)	0.112
Long-standing persistent atrial fibrillation	28 (22.6)	28 (22.4)	68 (27.4)	0.468
Hypertension	75 (60.5)	71 (56.8)	153 (61.7)	0.661
Diabetes mellitus	20 (16.1)	21 (16.8)	44 (17.7)	0.951
Dyslipidemia	60 (48.4)	61 (48.8)	106 (42.7)	0.430
Smoking history	83 (66.9)	77 (61.6)	142 (57.3)	0.192
Heart failure	24 (19.4)	27 (21.6)	41 (16.5)	0.465
Dilated cardiomyopathy	0 (0.0)	2 (1.6)	0 (0.0)	0.125
Hypertrophic cardiomyopathy	1 (0.8)	1 (0.8)	5 (2.0)	0.689
Sick sinus syndrome	2 (1.6)	2 (1.6)	3 (1.2)	1.000
Stroke or systemic thromboembolism	10 (8.1)	13 (10.4)	16 (6.5)	0.408
Sleep apnea syndrome	14 (11.3)	15 (12.0)	24 (9.7)	0.755
Thyroid disease	5 (4.0)	8 (6.4)	11 (4.4)	0.658
Chronic obstructive pulmonary disease	6 (4.8)	5 (4.0)	11 (4.4)	0.960
Liver disease	14 (11.3)	5 (4.0)	9 (3.6)	0.014
History of use of anti-arrhythmic drug	36 (29.0)	26 (20.8)	56 (22.6)	0.269
Hemoglobin	14.35 [13.47, 15.12]	14.50 [13.70, 15.30]	14.80 [13.80, 15.60]	0.036
Brain type natriuretic peptide	137.95 [87.65, 203.62]	148.75 [100.05, 237.62]	145.90 [99.25, 224.20]	0.465
Creatinine	0.86 [0.76, 1.00]	0.88 [0.78, 0.98]	0.88 [0.80, 1.01]	0.359
C-reactive protein	0.10 [0.06, 0.13]	0.10 [0.07, 0.20]	0.10 [0.06, 0.20]	0.602
Left ventricular ejection fraction	63.49 [59.34, 67.58]	63.27 [56.00, 68.03]	63.69 [57.64, 68.98]	0.654
Left atrial diameter	41.00 [38.00, 44.00]	42.30 [39.95, 46.00]	43.00 [39.00, 46.00]	0.006
Mitral regurgitation	0 (0.0)	4 (3.2)	6 (2.4)	0.145
Recurrence of atrial fibrillation	29 (23.4)	31 (24.8)	67 (27.0)	0.746

*Comparison among three groups.

**Table 4:** Patient characteristics in three datasets for each treatment branch.

	Training		Validation		Test		*P*-value*
	PVI-alone	PVI-plus	PVI-alone	PVI-plus	PVI-alone	PVI-plus	
N	63	61	62	63	124	124	
Age	68.00 [60.00, 72.50]	65.00 [58.00, 72.00]	66.00 [59.00, 72.75]	67.00 [60.00, 73.00]	68.00 [60.75, 73.00]	66.00 [58.00, 71.00]	0.421
Female sex	15 (23.8)	18 (29.5)	16 (25.8)	18 (28.6)	32 (25.8)	22 (17.7)	0.426
Body mass index	24.24 [21.74, 26.87]	23.32 [21.63, 26.02]	24.71 [22.24, 27.03]	24.77 [21.68, 26.40]	24.40 [22.14, 26.81]	24.21 [22.34, 26.43]	0.709
Family history of atrial fibrillation	9 (14.3)	4 (6.6)	5 (8.1)	7 (11.1)	9 (7.3)	4 (3.2)	0.110
Long-standing persistent atrial fibrillation	13 (20.6)	15 (24.6)	11 (17.7)	17 (27.0)	35 (28.2)	33 (26.6)	0.648
Hypertension	41 (65.1)	34 (55.7)	34 (54.8)	37 (58.7)	75 (60.5)	78 (62.9)	0.803
Diabetes mellitus	7 (11.1)	13 (21.3)	11 (17.7)	10 (15.9)	20 (16.1)	24 (19.4)	0.702
Dyslipidemia	30 (47.6)	30 (49.2)	32 (51.6)	29 (46.0)	50 (40.3)	56 (45.2)	0.740
Smoking history	46 (73.0)	37 (60.7)	39 (62.9)	38 (60.3)	68 (54.8)	74 (59.7)	0.302
Heart failure	12 (19.0)	12 (19.7)	13 (21.0)	14 (22.2)	21 (16.9)	20 (16.1)	0.890
Dilated cardiomyopathy	0 (0.0)	0 (0.0)	2 (3.2)	0 (0.0)	0 (0.0)	0 (0.0)	0.030
Hypertrophic cardiomyopathy	0 (0.0)	1 (1.6)	0 (0.0)	1 (1.6)	2 (1.6)	3 (2.4)	0.849
Sick sinus syndrome	1 (1.6)	1 (1.6)	1 (1.6)	1 (1.6)	1 (0.8)	2 (1.6)	0.990
Stroke or systemic thromboembolism	4 (6.3)	6 (9.8)	4 (6.5)	9 (14.3)	8 (6.5)	8 (6.5)	0.468
Sleep apnea syndrome	6 (9.5)	8 (13.1)	9 (14.5)	6 (9.5)	8 (6.5)	16 (12.9)	0.442
Thyroid disease	2 (3.2)	3 (4.9)	2 (3.2)	6 (9.5)	6 (4.8)	5 (4.0)	0.638
Chronic obstructive pulmonary disease	5 (7.9)	1 (1.6)	3 (4.8)	2 (3.2)	6 (4.8)	5 (4.0)	0.690
Liver disease	8 (12.7)	6 (9.8)	2 (3.2)	3 (4.8)	6 (4.8)	3 (2.4)	0.057
History of use of anti-arrhythmic drug	23 (36.5)	13 (21.3)	13 (21.0)	13 (20.6)	31 (25.0)	25 (20.2)	0.223
Hemoglobin	14.70 [13.35, 15.50]	14.20 [13.50, 14.90]	14.80 [14.03, 15.60]	14.20 [13.45, 14.90]	14.70 [13.57, 15.60]	14.90 [13.90, 15.60]	0.003
Brain type natriuretic peptide	143.65 [91.30, 200.45]	130.30 [86.47, 204.15]	132.20 [91.50, 208.10]	162.30 [104.70, 239.00]	142.70 [98.90, 223.60]	146.20 [101.20, 223.58]	0.670
Creatinine	0.88 [0.75, 0.97]	0.85 [0.77, 1.01]	0.89 [0.79, 0.99]	0.88 [0.76, 0.96]	0.86 [0.78, 1.03]	0.90 [0.82, 1.01]	0.579
C-reactive protein	0.10 [0.05, 0.13]	0.10 [0.08, 0.18]	0.10 [0.07, 0.19]	0.10 [0.07, 0.19]	0.10 [0.06, 0.20]	0.10 [0.06, 0.16]	0.758
Left ventricular ejection fraction	63.60 [59.88, 69.38]	63.37 [57.07, 66.39]	62.39 [55.04, 67.48]	64.04 [56.61, 68.61]	64.70 [59.88, 69.65]	63.11 [55.83, 67.71]	0.361
Left atrial diameter	41.00 [38.70, 44.00]	41.00 [37.10, 44.60]	41.60 [40.00, 44.20]	43.20 [39.90, 47.00]	43.00 [39.35, 46.00]	43.00 [39.00, 46.00]	0.023
Mitral regurgitation	0 (0.0)	0 (0.0)	0 (0.0)	4 (6.3)	2 (1.6)	4 (3.2)	0.087
Recurrence of atrial fibrillation	18 (28.6)	11 (18.0)	19 (30.6)	12 (19.0)	39 (31.5)	28 (22.6)	0.209

*Comparison among six groups.

## Results

### Study subjects and feature importance

**Figure 2:**
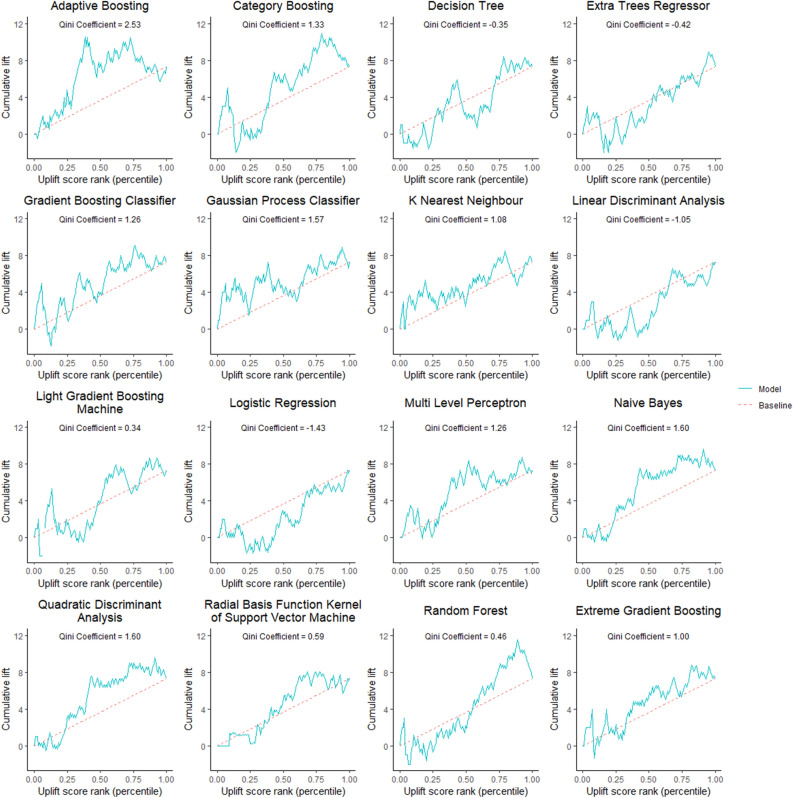
Qini curves of all models. The horizontal axis shows ranking of uplift score sorted in descending order, not uplift score in itself, and the vertical axis shows cumulative uplift.

**Figure 3:**
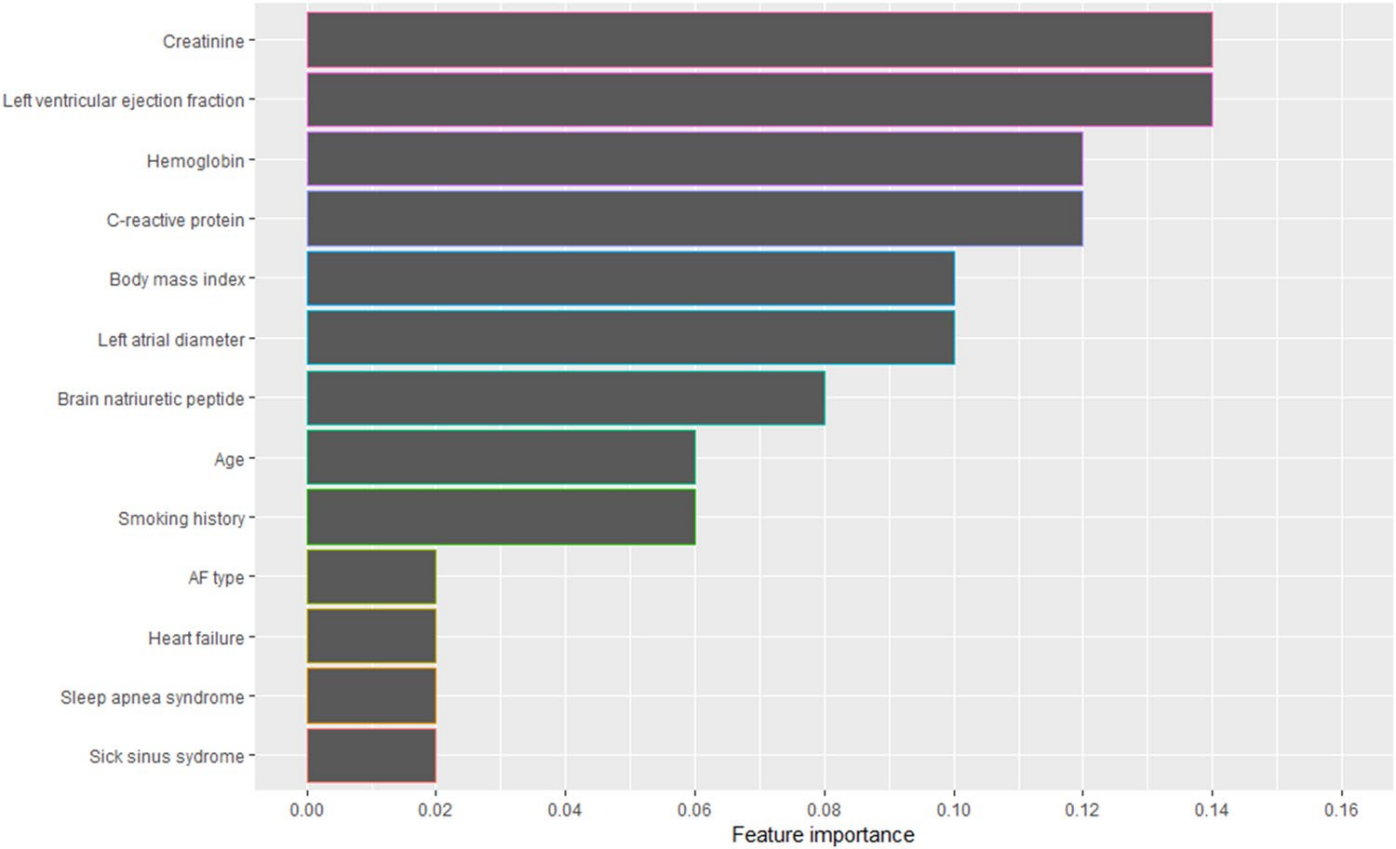
Feature importance of the top 13 variables.

A total of 512 patients were enrolled between March 2016 and September 2017. After excluding nine patients for protocol violation, five for errors in the electronic data collection system, and one for withdrawal of consent, 497 patients were analyzed in the EARNEST-PVI trial. Patient characteristic and outcome data in the training dataset used to train the models are shown in Table 1, and those used to plot the Qini curve are shown in Table 2. Qini curves of all models are shown in Fig. 2. We selected adaptive boosting to conduct predictions in the test dataset because this model showed the highest Qini coefficient among the 16 algorithms evaluated. Figure 3 shows the Gini importance of the top 13 variables. SHAP values are summarized in Supplementary information 1 (Supplementary Figure S1). Creatinine had the highest impact on prediction in the best model. We summarized patient characteristics of the overall cohort in Table 3 and those stratified by treatment arms in Table 4. The optimal uplift score cut-off according to the Qini curve was 0.0124. We divided the test dataset according to an uplift score of 0.0124 to obtain two groups: uplift score ≥ 0.0124 group (N = 116) and uplift score < 0.0124 group (N = 132) (Table 5). As shown in Table 5, patients with uplift score ≥ 0.0124 were mostly female, had lower frequency of smoking history and sleep apnea syndrome, and had lower hemoglobin and brain natriuretic peptide (BNP) levels than those with uplift score < 0.0124. Patient data based on actual allocation of treatment in the EARNEST-PVI trial are shown in Table 6. Supplementary Table S3 shows combination of procedure in in the training dataset used to train models, Supplementary Table S4 shows combination of procedure in the training dataset used to plot Qini curves, Supplementary Table S5 shows combination of procedure in Uplift score ≥ 0.0124 group in the test dataset, and Supplementary Table S6 shows combination of procedure in Uplift score < 0.0124 group in the test dataset (Supplementary information 2).

**Table 5:** Patient characteristics in the test dataset.

	Uplift score ≥ 0.0124	Uplift score < 0.0124	*P*-value
n	116	132	
Age	68.00 [59.75, 73.00]	66.00 [59.00, 70.00]	0.393
Female sex	37 (31.9)	17 (12.9)	< 0.001
Body mass index	24.17 [22.29, 25.76]	24.45 [22.30, 27.15]	0.305
Family history of atrial fibrillation	6 (5.2)	7 (5.3)	> 0.999
Long-standing persistent atrial fibrillation	35 (30.2)	33 (25.0)	0.394
Hypertension	75 (64.7)	78 (59.1)	0.432
Diabetes mellitus	20 (17.2)	24 (18.2)	0.869
Dyslipidemia	48 (41.4)	58 (43.9)	0.701
Smoking history	41 (35.3)	101 (76.5)	< 0.001
Heart failure	24 (20.7)	17 (12.9)	0.123
Dilated cardiomyopathy	0 (0.0)	0 (0.0)	NA
Hypertrophic cardiomyopathy	2 (1.7)	3 (2.3)	> 0.999
Sick sinus syndrome	3 (2.6)	0 (0.0)	0.101
Stroke or systemic thromboembolism	9 (7.8)	7 (5.3)	0.451
Sleep apnea syndrome	3 (2.6)	21 (15.9)	< 0.001
Thyroid disease	4 (3.4)	7 (5.3)	0.549
Chronic obstructive pulmonary disease	6 (5.2)	5 (3.8)	0.759
Liver disease	2 (1.7)	7 (5.3)	0.180
History of use of anti-arrhythmic drug	22 19.0)	34 (25.8)	0.225
Hemoglobin	14.40 [13.40, 15.10]	15.20 [14.38, 16.00]	< 0.001
Brain type natriuretic peptide	138.15 [90.62, 178.90]	152.90 [103.20, 252.50]	0.017
Creatinine	0.85 [0.75, 1.05]	0.90 [0.82, 0.98]	0.199
C-reactive protein	0.10 [0.06, 0.19]	0.10 [0.06, 0.20]	0.488
Left ventricular ejection fraction	63.87 [56.63, 71.16]	63.27 [58.98, 68.69]	0.950
Left atrial diameter	44.00 [39.00, 46.40]	42.45 [39.58, 45.00]	0.063
Mitral regurgitation	5 (4.3)	1 (0.8)	0.101
Recurrence of atrial fibrillation	31 (23.5)	36 (31.0)	0.199

**Table 6:** Patient characteristics in the test dataset according to uplift score and allocation of procedure.

	Uplift score ≥ 0.0124			Uplift score < 0.0124		
	PVI-alone	PVI-plus	*P*-value	PVI-alone	PVI-plus	*P*-value
n	64	52		60	72	
Age	69.00 [60.75, 73.00]	66.50 [57.50, 73.00]	0.432	67.00 [60.75, 73.00]	65.50 [58.75, 70.00]	0.069
Female sex	22 (34.4)	15 (28.8)	0.554	10 (16.7)	7 (9.7)	0.299
Body mass index	24.42 [22.16, 25.81]	23.98 [22.34, 25.06]	0.585	24.37 [22.12, 27.49]	24.52 [22.40, 26.50]	0.996
Family history of atrial fibrillation	4 (6.2)	2 (3.8)	0.690	5 (8.3)	2 (2.8)	0.244
Long-standing persistent atrial fibrillation	20 (31.2)	15 (28.8)	0.840	15 (25.0)	18 (25.0)	> 0.999
Hypertension	42 (65.6)	33 (63.5)	0.847	33 (55.0)	45 (62.5)	0.477
Diabetes mellitus	8 (12.5)	12 (23.1)	0.147	12 (20.0)	12 (16.7)	0.656
Dyslipidemia	23 (35.9)	25 (48.1)	0.255	27 (45.0)	31 (43.1)	0.861
Smoking history	23 (35.9)	18 (34.6)	> 0.999	45 (75.0)	56 (77.8)	0.837
Heart failure	11 (17.2)	13 (25.0)	0.360	10 (16.7)	7 (9.7)	0.299
Dilated cardiomyopathy	0 (0.0)	0 (0.0)	NA	0 (0.0)	0 (0.0)	NA
Hypertrophic cardiomyopathy	0 (0.0)	2 (3.8)	0.199	2 (3.3)	1 (1.4)	0.591
Sick sinus syndrome	1 (1.6)	2 (3.8)	0.586	0 (0.0)	0 (0.0)	NA
Stroke or systemic thromboembolism	5 (7.8)	4 (7.7)	> 0.999	3 (5.0)	4 (5.6)	> 0.999
Sleep apnea syndrome	1 (1.6)	2 (3.8)	0.586	7 (11.7)	14 (19.4)	0.243
Thyroid disease	1 (1.6)	3 (5.8)	0.324	5 (8.3)	2 (2.8)	0.244
Chronic obstructive pulmonary disease	4 (6.2)	2 (3.8)	0.690	2 (3.3)	3 (4.2)	> 0.999
Liver disease	2 (3.1)	0 (0.0)	0.501	4 (6.7)	3 (4.2)	0.701
History of use of anti-arrhythmic drug	14 (21.9)	8 (15.4)	0.477	17 (28.3)	17 (23.6)	0.555
Hemoglobin	14.20 [13.20, 15.12]	14.60 [13.60, 15.10]	0.543	15.40 [14.35, 16.00]	15.00 [14.38, 16.00]	0.638
Brain type natriuretic peptide	132.45 [90.88, 169.28]	143.35 [88.78, 218.88]	0.625	151.90 [101.70, 249.60]	155.40 [105.75, 252.68]	0.902
Creatinine	0.82 [0.71, 1.05]	0.90 [0.78, 1.06]	0.101	0.90 [0.81, 1.03]	0.90 [0.83, 0.97]	0.633
C-reactive protein	0.10 [0.05, 0.14]	0.10 [0.08, 0.22]	0.158	0.10 [0.06, 0.21]	0.08 [0.05, 0.13]	0.048
Left ventricular ejection fraction	65.50 [59.90, 71.26]	62.39 [52.47, 68.76]	0.064	63.22 [59.72, 68.95]	63.27 [57.63, 67.51]	0.499
Left atrial diameter	45.00 [39.00, 47.00]	43.00 [39.00, 46.00]	0.356	42.45 [40.00, 45.00]	42.40 [39.00, 45.00]	0.965
Mitral regurgitation	1 (1.6)	4 (7.7)	0.172	1 (1.7)	0 (0.0)	0.455
Recurrence of atrial fibrillation	26 (40.6)	10 (19.2)	0.016	13 (21.7)	18 (25.0)	0.685

### Clinical endpoints

**Figure 4:**
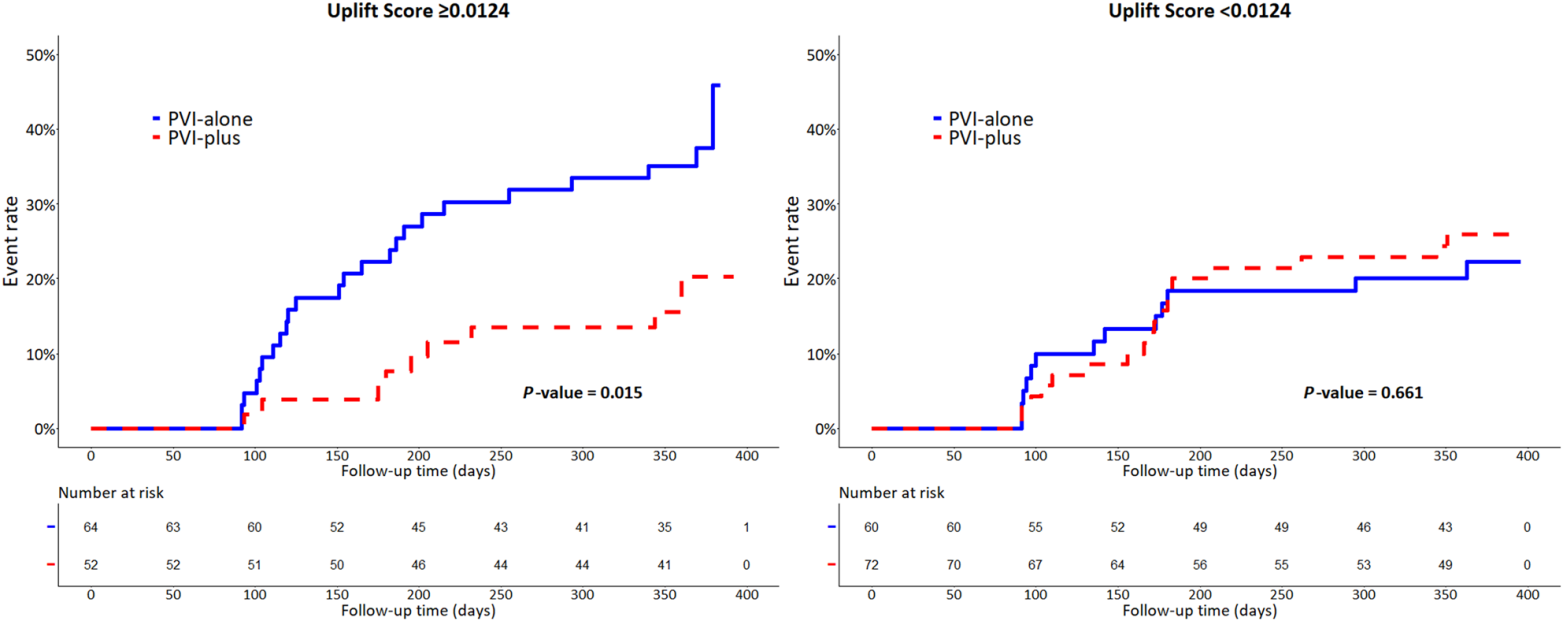
Kaplan–Meier analysis with a log-rank test of recurrence of atrial fibrillation in patients with uplift score ≥ 0.0124 (left) and uplift score < 0.0124 (right) in test dataset. PVI: pulmonary vein isolation.

**Figure 5:**
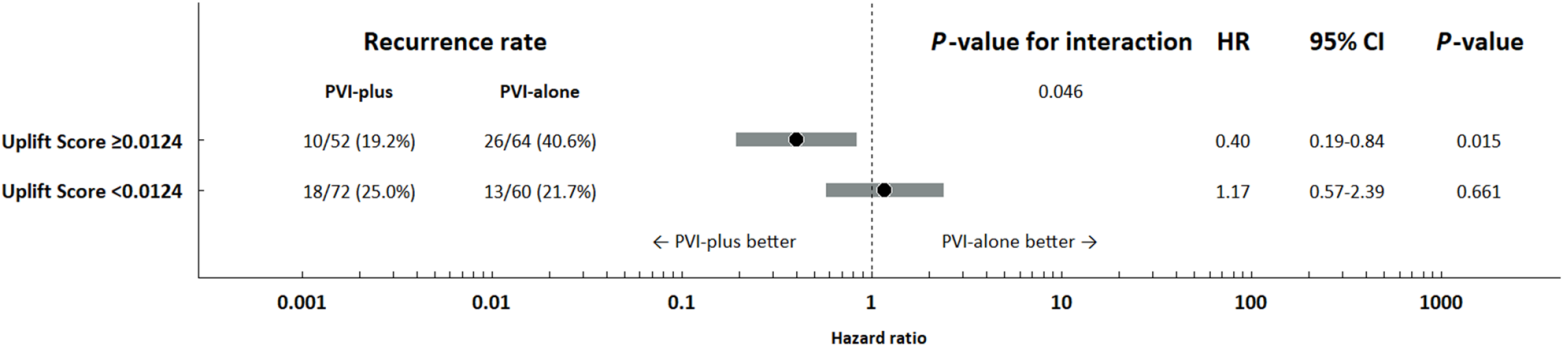
Hazard ratio of the primary endpoint using a Cox proportional hazards model in test dataset. PVI: pulmonary vein isolation; HR: hazard ratio; 95% CI: 95% confidence interval.

Figure 4 shows the results of Kaplan–Meier analysis for the primary endpoint in test dataset. Among patients with uplift score ≥ 0.0124, the event rate of recurrence of AF was significantly lower in those who received PVI-plus than those who received PVI-alone in the test dataset (PVI-plus [10/52, 19.2%] vs PVI-alone [26/64, 40.6%], HR 0.40; 95% CI 0.19–0.84; *P*-value = 0.015). In contrast, among patients with uplift score < 0.0124, no significant differences were observed in the event rate of recurrence of AF between PVI-plus and PVI-alone in the test dataset (PVI-plus [18/72, 25.0%] vs PVI-alone [13/60, 21.7%], HR 1.17; 95% CI 0.57–2.39; *P*-value: 0.661). There was a significant interaction between uplift score and treatment (*P*-value for interaction: 0.046) (Fig. 5).

## Discussion

### Main findings

Our study has revealed the utility of uplift modeling in identifying a subset of patients with persistent AF who would most benefit from extensive ablation. By Using an adaptive boosting model on data from the EARNEST-PVI trial, we found that an extensive ablation strategy, such as linear ablation and/or CFAE ablation in addition to PVI, was efficacious in patients with uplift score ≥ 0.0124, but demonstrated similar efficacy to PVI-alone in those with uplift score < 0.0124. These results imply that calculating the uplift score using the 26 variables identified in this study (supplemental material) and stratifying patients based on an uplift score threshold of 0.0124 may be a promising approach for selecting the most appropriate ablation strategy for patients with persistent AF.

### Strength of this study

This study presents the initial evidence that uplift modeling via machine learning is a valuable tool for identifying patients with persistent atrial fibrillation who may benefit from extensive catheter ablation or for whom pulmonary vein isolation-alone is adequate for rhythm control. Furthermore, it shows that an uplift score of 0.0124 derived from our model is an effective threshold for distinguishing between those who will or will not benefit from extensive catheter ablation. Several randomized controlled trials have been unable to show the superiority of extensive catheter ablation strategies over PVI-alone in persistent AF patients^6,16^. A meta-analysis on the efficacy of extensive catheter ablation, including CFAE and linear ablation, reported that there were no significant differences for maintaining sinus rhythm between PVI with extensive catheter ablation and PVI-alone^17^. Further, superiority of PVI-plus over PVI-alone could not be established also in the EARNEST-PVI trial. These inconclusive results related to the superiority of PVI-plus over PVI-alone might be attributed to the heterogeneity of patients with persistent AF, and suggest the importance of administering the appropriate treatment to the appropriate patients among those with persistent AF. The present study proposes a novel, useful approach, which employs uplift modeling to stratify persistent AF patients into those who may benefit from extensive ablation and those who may not require more than PVI-alone, has the potential to reduce unnecessary costs and complications. Further prospective studies are needed to confirm the clinical applicability of this approach and the factors we have identified for determining appropriate catheter ablation strategies.

### Stratification of persistent atrial fibrillation

This study is the first report to employ uplift modeling for identifying a particular subgroup of patients with persistent AF who may derive benefits from extensive ablation, such as linear ablation or CFAE ablation, in addition to PVI, to maintain sinus rhythm. Several previous investigations have reported predictors of AF recurrence after catheter ablation, regardless of extensive ablation, including left atrial size, type of AF, AF duration, female gender, and machine learning models^18–20^. Nevertheless, only a few studies have been aimed at identifying a specific patient group that requires additional extensive ablation among those with persistent AF. We previously reported possible stratification by sex and DR-FLASH score^9,10^. We observed that PVI-plus presented a lower risk of AF recurrence than PVI-alone in patients with a DR-FLASH score of > 3, suggesting that the DR-FLASH score is a valuable tool for identifying patients with persistent AF who will benefit from PVI-plus^10^. In this novel study, we utilized a novel machine learning approach to identify patients who may benefit from extensive ablation by testing 16 probabilistic classification models using uplift modeling with 26 clinical factors. Through adaptive boosting, we obtained a hazard ratio of 0.40 (95% CI 0.19–0.84) for PVI-plus compared to PVI-alone in patients with an uplift score ≥ 0.0124, which was lower than that observed in patients with DR-FLASH score > 3 (HR 0.45, 95% CI 0.28–0.72) in our previous study^10^. Moreover, the clinical factors employed in this study can be non-invasively obtained via medical interview, blood examination, and transthoracic echocardiography. These results suggest that uplift modeling can be a valuable noninvasive tool to improve our ability to identify patients who require extensive ablation in addition to PVI among those with persistent AF. Nevertheless, further prospective studies may be warranted to assess the superior strategy between the DR-FLASH score and uplift modeling. Additionally, efforts to reduce the number of factors required for uplift modeling while maintaining discrimination power will be pivotal to enhance the practicality of this model in daily clinical practice. The uplift score is more difficult to implement in the clinical setting because our uplift modeling requires 26 variables and a substantial technical skillset. Nevertheless, the reason why we performed uplift modeling is because we were motivated to comprehensively analyze the dataset of the EARNEST-PVI trial. The present study on stratification with uplift modeling is data-driven research, whereas the previous study on stratification with DR-FLASH score is theory-driven research. Although hazard ratios in both studies appear almost identical in result, stratification by the uplift modeling was more accurate than by the DR-FLASH score. It is meaningful to reveal usefulness of uplift modeling for strictly selecting a catheter ablation strategy.

### Characteristics of patients with high uplift score

The clinical factors that contribute to a high uplift score using an adaptive boosting strategy offer insight into the characteristics of patients who would benefit from extensive ablation. Our adaptive boosting approach identified serum creatinine level, left ventricular ejection fraction, hemoglobin level, BNP, C-reactive protein (CRP), left atrial diameter, smoking history, body mass index, history of heart failure and sleep apnea syndrome as the top ten factors with high feature importance (Fig. 3). Although all of these factors have already been reported to be predictors of AF recurrence after catheter ablation^21,22^, we were unable to determine the exact relationship between these variables and the uplift score due to the non-linear nature of machine learning. We therefore conducted a comparison of patient characteristics between those who had an uplift score ≥ 0.0124 and those who had a score < 0.0124 to assess the effect of each factor on the uplift score (Table 5). The analysis revealed that patients with an uplift score greater ≥ 0.0124 were predominantly female, had lower frequencies of smoking history and sleep apnea syndrome, and had lower hemoglobin and BNP levels, as well as larger left atrial diameters (Table 5). These observations suggest that these features may contribute to a high uplift score. Furthermore, given that female sex^23^, lower hemoglobin^24^ and larger left atrial size^25,26^ are already known to be associated with arrhythmogenic substrate, which can cause AF recurrence, these findings suggest that patients with arrhythmogenic substrate would benefit from extensive ablation. In contrast, patients with high uplift score also had lower frequencies of smoking history^27^ and sleep apnea^28^, and lower BNP levels^29^, which are generally considered to be associated with lower risk of recurrence after catheter ablation. While the true reasons for this discrepancy are unknown, one possible explanation is that machine learning using an adaptive boosting strategy led to the identification of patients who would benefit from extensive ablation based on different criteria from those previously reported, such as the presence of arrhythmogenic substrate. Another possible explanation is that smoking, sleep apnea and high BNP are less strongly associated with arrhythmogenic substrate than factors like female sex, lower hemoglobin and larger atrial size. Given that uplift modeling is used to identify patients who would benefit the most from an intervention, rather than to predict recurrence of an event, these results suggest that extensive ablation may not be effective at all for patients with smoking, sleep apnea and high BNP. Nevertheless, these findings suggest that the uplift score and machine learning may be useful for identifying a specific population that would benefit from extensive ablation, and that there may exist previously unrecognized criteria or algorithms that could enhance our ability to identify such a population.

### Limitations

Several limitations exist in the current study. Firstly, the techniques employed for additional left atrial ablation in the PVI-plus category of the EARNEST-PVI trial were not pre-specified, resulting in heterogeneity. The trial was initially designed to investigate the non-inferiority of PVI-alone against any extensive catheter ablation for patients with persistent AF. Secondly, the study was conducted solely in an East Asian population, thus limiting the generalizability of the findings to other ethnic groups. Thirdly, the primary endpoint, recurrence of AF, may have been underestimated since only regular 12-lead ECG and Holter ECG were employed at each visit, while event recorders or implantable devices were not utilized to detect recurrence. Fourth, the uplift score is difficult to implement in the clinical setting because our uplift modeling requires 26 variables plus an advanced degree to calculate. Finally, all the included patients underwent their first procedure and therefore results are not applicable to redo procedures as is so often the case with persistent AF patients.

## Conclusions

We demonstrated that the application of machine learning using uplift modeling can be useful for identifying a specific subgroup of patients with persistent AF who would most benefit from an extensive ablation strategy, comprising linear ablation and/or CFAE ablation in addition to PVI. An uplift score of 0.0124, calculated using our model, may be a useful threshold for stratifying patients with persistent AF who do and do not require extensive ablation in addition to PVI. However, additional prospective investigations are necessary to determine the efficacy of this approach.

### Supplementary Information

Supplementary Information 1.

Supplementary Information 2.

Supplementary Information 3.

## Data Availability

The datasets generated and/or analysed during the current study are not publicly available due to institutional review board restrictions, but are available from the corresponding author on reasonable request.

## References

[1] Rubin DB (1974). Estimating causal effects of treatments in randomized and nonrandomized studies. J. Educ. Psychol..

[2] Jaskowski, M, Jaroszewicz, S. Uplift modeling for clinical trial data. Paper/Poster presented 2012.

[3] Hindricks G, Potpara T, Dagres N, Arbelo E, Bax JJ, Blomstrom-Lundqvist C, Boriani G, Castella M, Dan GA, Dilaveris PE (2020). 2020 ESC Guidelines for the diagnosis and management of atrial fibrillation developed in collaboration with the European Association of Cardio-Thoracic Surgery (EACTS). Eur. Heart J..

[4] January CT, Wann LS, Calkins H, Chen LY, Cigarroa JE, Cleveland JC, Ellinor PT, Ezekowitz MD, Field ME, Furie KL (2019). 2019 AHA/ACC/HRS focused update of the 2014 AHA/ACC/HRS guideline for the management of patients with atrial fibrillation: A report of the american college of cardiology/american heart association task force on clinical practice guidelines and the Heart Rhythm Society in Collaboration With the Society of Thoracic Surgeons. Circulation..

[5] Murakawa Y, Yamane T, Goya M, Inoue K, Naito S, Kumagai K, Miyauchi Y, Morita N, Nogami A, Shoda M (2018). Influence of substrate modification in catheter ablation of atrial fibrillation on the incidence of acute complications: Analysis of 10 795 procedures in J-CARAF Study 2011–2016. J Arrhythm..

[6] Verma A, Jiang CY, Betts TR, Chen J, Deisenhofer I, Mantovan R, Macle L, Morillo CA, Haverkamp W, Weerasooriya R (2015). Approaches to catheter ablation for persistent atrial fibrillation. N Engl J Med..

[7] Inoue K, Hikoso S, Masuda M, Furukawa Y, Hirata A, Egami Y, Watanabe T, Minamiguchi H, Miyoshi M, Tanaka N (2020). Pulmonary vein isolation alone vs. more extensive ablation with defragmentation and linear ablation of persistent atrial fibrillation: The EARNEST-PVI trial. Europace..

[8] Jadidi AS, Lehrmann H, Keyl C, Sorrel J, Markstein V, Minners J, Park CI, Denis A, Jais P, Hocini M (2016). Ablation of persistent atrial fibrillation targeting low-voltage areas with selective activation characteristics. Circ. Arrhythm Electrophysiol..

[9] Sato T, Sotomi Y, Hikoso S, Nakatani D, Mizuno H, Okada K, Dohi T, Kitamura T, Sunaga A, Kida H (2021). Sex differences in the efficacy of pulmonary vein isolation alone vs. extensive catheter ablation in patients with persistent atrial fibrillation. Circ J..

[10] Sato T, Sotomi Y, Hikoso S, Nakatani D, Mizuno H, Okada K, Dohi T, Kitamura T, Sunaga A, Kida H (2022). DR-FLASH score is useful for identifying patients with persistent atrial fibrillation who require extensive catheter ablation procedures. J Am Heart Assoc..

[11] Rolf S, Kircher S, Arya A, Eitel C, Sommer P, Richter S, Gaspar T, Bollmann A, Altmann D, Piedra C (2014). Tailored atrial substrate modification based on low-voltage areas in catheter ablation of atrial fibrillation. Circ Arrhythm Electrophysiol..

[12] Dohi T, Nakatani D, Inoue K, Hikoso S, Oka T, Hayashi K, Masuda M, Furukawa Y, Kawasaki M, Egami Y (2019). Effect of extensive ablation on recurrence in patients with persistent atrial fibrillation treated with pulmonary vein isolation (EARNEST-PVI) trial: Design and rationale. J Cardiol..

[13] Inoue K, Sotomi Y, Masuda M, Furukawa Y, Hirata A, Egami Y, Watanabe T, Minamiguchi H, Miyoshi M, Tanaka N (2021). Efficacy of extensive ablation for persistent atrial fibrillation with trigger-based vs. substrate-based mechanisms: A prespecified subanalysis of the EARNEST-PVI Trial. Circ J..

[14] Menze BH, Kelm BM, Masuch R, Himmelreich U, Bachert P, Petrich W, Hamprecht FA (2009). A comparison of random forest and its Gini importance with standard chemometric methods for the feature selection and classification of spectral data. BMC Bioinform..

[15] Rodriguez-Perez R, Bajorath J (2020). Interpretation of machine learning models using shapley values: application to compound potency and multi-target activity predictions. J Comput Aided Mol Des..

[16] Vogler J, Willems S, Sultan A, Schreiber D, Luker J, Servatius H, Schaffer B, Moser J, Hoffmann BA, Steven D (2015). Pulmonary vein isolation versus defragmentation: The CHASE-AF clinical trial. J Am Coll Cardiol..

[17] Scott PA, Silberbauer J, Murgatroyd FD (2016). The impact of adjunctive complex fractionated atrial electrogram ablation and linear lesions on outcomes in persistent atrial fibrillation: a meta-analysis. Europace..

[18] Zhou X, Nakamura K, Sahara N, Takagi T, Toyoda Y, Enomoto Y, Hara H, Noro M, Sugi K, Moroi M (2022). Deep learning-based recurrence prediction of atrial fibrillation after catheter ablation. Circ J..

[19] Furui K, Morishima I, Morita Y, Kanzaki Y, Takagi K, Yoshida R, Nagai H, Watanabe N, Yoshioka N, Yamauchi R (2020). Predicting long-term freedom from atrial fibrillation after catheter ablation by a machine learning algorithm: Validation of the CAAP-AF score. J Arrhythm..

[20] Atta-Fosu T, LaBarbera M, Ghose S, Schoenhagen P, Saliba W, Tchou PJ, Lindsay BD, Desai MY, Kwon D, Chung MK (2021). A new machine learning approach for predicting likelihood of recurrence following ablation for atrial fibrillation from CT. BMC Med Imaging..

[21] Epicoco, G., Sorgente, A.. Predictors of atrial fibrillation recurrence after catheter ablation. *J Atr Fibrillation.* **6**, 1016. 10.4022/jafib.1016 (2014). 10.4022/jafib.1016 PMC4956137 27957049

[22] Cheng WH, Lo LW, Lin YJ, Chang SL, Hu YF, Hung Y, Chung FP, Chang TY, Huang TC, Yamada S (2018). Cigarette smoking causes a worse long-term outcome in persistent atrial fibrillation following catheter ablation. J Cardiovasc Electrophysiol..

[23] Akoum N, Mahnkopf C, Kholmovski EG, Brachmann J, Marrouche NF (2018). Age and sex differences in atrial fibrosis among patients with atrial fibrillation. Europace..

[24] Xu D, Murakoshi N, Sairenchi T, Irie F, Igarashi M, Nogami A, Tomizawa T, Yamaguchi I, Yamagishi K, Iso H (2015). Anemia and reduced kidney function as risk factors for new onset of atrial fibrillation (from the Ibaraki prefectural health study). Am J Cardiol..

[25] Chang SL, Tai CT, Lin YJ, Wongcharoen W, Lo LW, Lee KT, Chang SH, Tuan TC, Chen YJ, Hsieh MH (2007). The role of left atrial muscular bundles in catheter ablation of atrial fibrillation. J Am Coll Cardiol..

[26] Sanders P, Morton JB, Davidson NC, Spence SJ, Vohra JK, Sparks PB, Kalman JM (2003). Electrical remodeling of the atria in congestive heart failure: electrophysiological and electroanatomic mapping in humans. Circulation..

[27] Shan H, Zhang Y, Lu Y, Zhang Y, Pan Z, Cai B, Wang N, Li X, Feng T, Hong Y (2009). Downregulation of miR-133 and miR-590 contributes to nicotine-induced atrial remodelling in canines. Cardiovasc Res..

[28] Nalliah CJ, Wong GR, Lee G, Voskoboinik A, Kee K, Goldin J, Watts T, Linz D, Wirth D, Parameswaran R (2021). Sleep apnoea has a dose-dependent effect on atrial remodelling in paroxysmal but not persistent atrial fibrillation: A high-density mapping study. Europace..

[29] Iwanaga Y, Nishi I, Furuichi S, Noguchi T, Sase K, Kihara Y, Goto Y, Nonogi H (2006). B-type natriuretic peptide strongly reflects diastolic wall stress in patients with chronic heart failure: Comparison between systolic and diastolic heart failure. J Am Coll Cardiol..

