# Supplementary material for: Uplift modeling to identify patients who require extensive catheter ablation procedures among patients with persistent atrial fibrillation
Source: Sci Rep. 2024 Feb 1;14:2634. doi: 10.1038/s41598-024-52976-7 (PMC10834528; doi:10.1038/s41598-024-52976-7)

Supplementary Figure 1. SHapley Additive exPlanations (SHAP) for feature importance in three cohorts

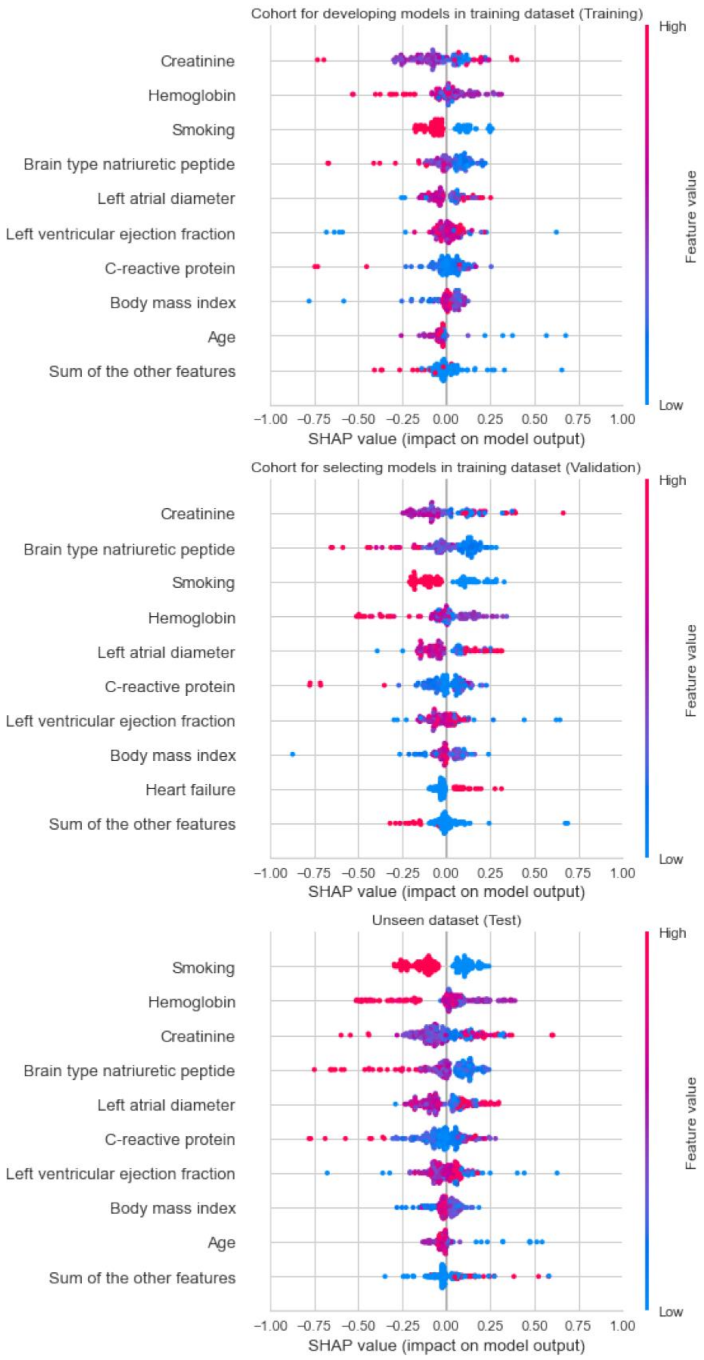

Supplement: Supplementary file 1 — Supplementary Information 1. [file 41598_2024_52976_MOESM1_ESM.pdf]
