# Supplementary material for: Uplift modeling to identify patients who require extensive catheter ablation procedures among patients with persistent atrial fibrillation
Source: Sci Rep. 2024 Feb 1;14:2634. doi: 10.1038/s41598-024-52976-7 (PMC10834528; doi:10.1038/s41598-024-52976-7)
Supplement: Supplementary file 2 — Supplementary Information 2. [file 41598_2024_52976_MOESM2_ESM.pdf]

Supplementary information

Supplementary Table 1. A total of 53 variables as candidates in the present analysis

	variable type	missing
Age	numerical	0.0%
Female sex	categorical	0.0%
Height	numerical	0.0%
Weight	numerical	0.0%
Body mass index	numerical	0.0%
Family history of atrial fibrillation	categorical	0.0%
Long-standing persistent atrial fibrillation	categorical	0.0%
Duration of atrial fibrillation	numerical	0.0%
Period from diagnosis of atrial fibrillation	numerical	0.0%
Hypertension	categorical	0.0%
Diabetes mellitus	categorical	0.0%
Dyslipidemia	categorical	0.0%
Smoking history	categorical	0.0%
Heart failure	categorical	0.0%
Dilated cardiomyopathy	categorical	0.0%
Hypertrophic cardiomyopathy	categorical	0.0%
Vascular disease	categorical	0.0%
Prior myocardial infarction	categorical	0.0%
Angina pectoris	categorical	0.0%
History of percutaneous cardiovascular intervention	categorical	0.0%
Peripheral arterial disease	categorical	3.2%
Plaque of aorta	categorical	0.0%
Valvular disease	categorical	9.6%
Sick sinus syndrome	categorical	0.0%
History of stroke or systemic thromboembolism	categorical	0.0%
History of stroke	categorical	0.0%
History of transient ischemic attack	categorical	0.0%
Systemic thromboembolism	categorical	0.0%
Sleep apnea syndrome	categorical	0.0%
Thyroid disease	categorical	0.0%
Chronic obstructive pulmonary disease	categorical	0.0%
Liver disease	categorical	0.0%

History of use of anti-arrhythmic drug	categorical	0.0%
Hemoglobin	numerical	0.0%
Brain-type natriuretic peptide	numerical	3.2%
Creatinine	numerical	0.0%
C-reactive protein	numerical	9.6%
Left ventricular end-diastolic diameter	numerical	0.0%
Left ventricular end-systolic diameter	numerical	0.0%
Interventricular septum wall thickness	numerical	0.0%
Left ventricular posterior wall thickness	numerical	0.0%
Left ventricular ejection fraction	numerical	0.0%
Left atrial diameter	numerical	0.0%
Mitral regurgitation	categorical	0.0%
Heart rate	numerical	0.0%
Anticoagulation	categorical	0.0%
Antiplatelet	categorical	0.0%
Angiotensin-converting enzyme inhibitor	categorical	0.0%
Angiotensin receptor blocker	categorical	0.0%
Calcium channel blocker	categorical	0.0%
Beta blocker	categorical	0.0%
Diuretics	categorical	0.0%
Mitral regurgitation	categorical	0.0%

Supplementary Table 2. A total of 26 variables finally included in the present analysis

	variable type	missing
Age	numerical	0.0%
Female sex	categorical	0.0%
Body mass index	numerical	0.0%
Family history of atrial fibrillation	categorical	0.0%
Long-standing persistent atrial fibrillation	categorical	0.0%
Hypertension	categorical	0.0%
Diabetes mellitus	categorical	0.0%
Dyslipidemia	categorical	0.0%
Smoking history	categorical	0.0%
Heart failure	categorical	0.0%
Dilated cardiomyopathy	categorical	0.0%
Hypertrophic cardiomyopathy	categorical	0.0%
Sick sinus syndrome	categorical	0.0%
Stroke or systemic thromboembolism	categorical	0.0%
Sleep apnea syndrome	categorical	0.0%
Thyroid disease	categorical	0.0%
Chronic obstructive pulmonary disease	categorical	0.0%
Liver disease	categorical	0.0%
History of use of anti-arrhythmic drug	categorical	0.0%
Hemoglobin	numerical	0.0%
Brain-type natriuretic peptide	numerical	3.2%
Creatinine	numerical	0.0%
C-reactive protein	numerical	9.6%
Left ventricular ejection fraction	numerical	0.0%
Left atrial diameter	numerical	0.0%
Mitral regurgitation	categorical	0.0%

Supplementary Table 3. Combination of procedure in in the training dataset used to train models

	PVI-alone	PVI-plus
N	63	61
procedure combination (%)		
CFAE	0 (0.0)	6 (9.8)
Roof line + Anterior line	0 (0.0)	14 (23.0)
Roof line + Bottom line + CFAE	0 (0.0)	1 (1.6)
Roof line + Anterior line + Bottom line	0 (0.0)	5 (8.2)
Roof line + Anterior line + Bottom line + Mitral isthmus line	0 (0.0)	1 (1.6)
Roof line + Bottom line + Mitral isthmus line	0 (0.0)	16 (26.2)
Roof line + Mitral isthmus line	0 (0.0)	18 (29.5)
PVI-alone	63 (100.0)	0 (0.0)

Supplementary Table 4. Combination of procedure in the training dataset used to plot Qini curves

	PVI-alone	PVI-plus
N	62	63
procedure combination (%)		
CFAE	0 (0.0)	5 (7.9)
Roof line + CFAE	0 (0.0)	1 (1.6)
Roof line + Anterior line	0 (0.0)	13 (20.6)
Roof line + Anterior line + Mitral isthmus line	0 (0.0)	1 (1.6)
Roof line + Bottom line + CFAE	0 (0.0)	1 (1.6)
Roof line + Anterior line + Bottom line + Mitral isthmus line	0 (0.0)	2 (3.2)
Roof line + Bottom line + Mitral isthmus line	0 (0.0)	29 (46.0)
Roof line + Mitral isthmus line	0 (0.0)	8 (12.7)
No additional procedures	0 (0.0)	3 (4.8)
PVI-alone	62 (100.0)	0 (0.0)

Supplementary Table 5. Combination of procedure in Uplift score ≥ 0.0124 group in the test dataset

	PVI-alone	PVI-plus
N	64	52
procedure combination (%)		
CFAE	0 (0.0)	13 (25.0)
Anterior line + Mitral isthmus line	0 (0.0)	1 (1.9)
Roof line + Bottom line + Mitral isthmus line	0 (0.0)	20 (38.5)
Roof line + Mitral isthmus line	0 (0.0)	18 (34.6)
PVI-alone	64 (100.0)	0 (0.0)

Supplementary Table 6. Combination of procedure in Uplift score < 0.0124 group in the test dataset

	PVI-alone	PVI-plus
N	60	72
procedure combination (%)		
CFAE	0 (0.0)	10 (13.9)
Roof line + CFAE	0 (0.0)	1 (1.4)
Roof line + Anterior line	0 (0.0)	3 (4.2)
Roof line + Bottom line	0 (0.0)	1 (1.4)
Roof line + Bottom line + Mitral isthmus line	0 (0.0)	34 (47.2)
Roof line + Mitral isthmus line	0 (0.0)	23 (31.9)
PVI-alone	60 (100.0)	0 (0.0)

Uplift Modeling on a Dataset

We have included files for performing uplift score calculations in the "Supplementary materials" folder.
